# Supplementary figures and images for: Immunogenicity evaluation of MS2 phage-mediated chimeric nanoparticle displaying an immunodominant B cell epitope of foot-and-mouth disease virus
Source: PeerJ. 2018 May 23;6:e4823. doi: 10.7717/peerj.4823 (PMC5970553; doi:10.7717/peerj.4823)

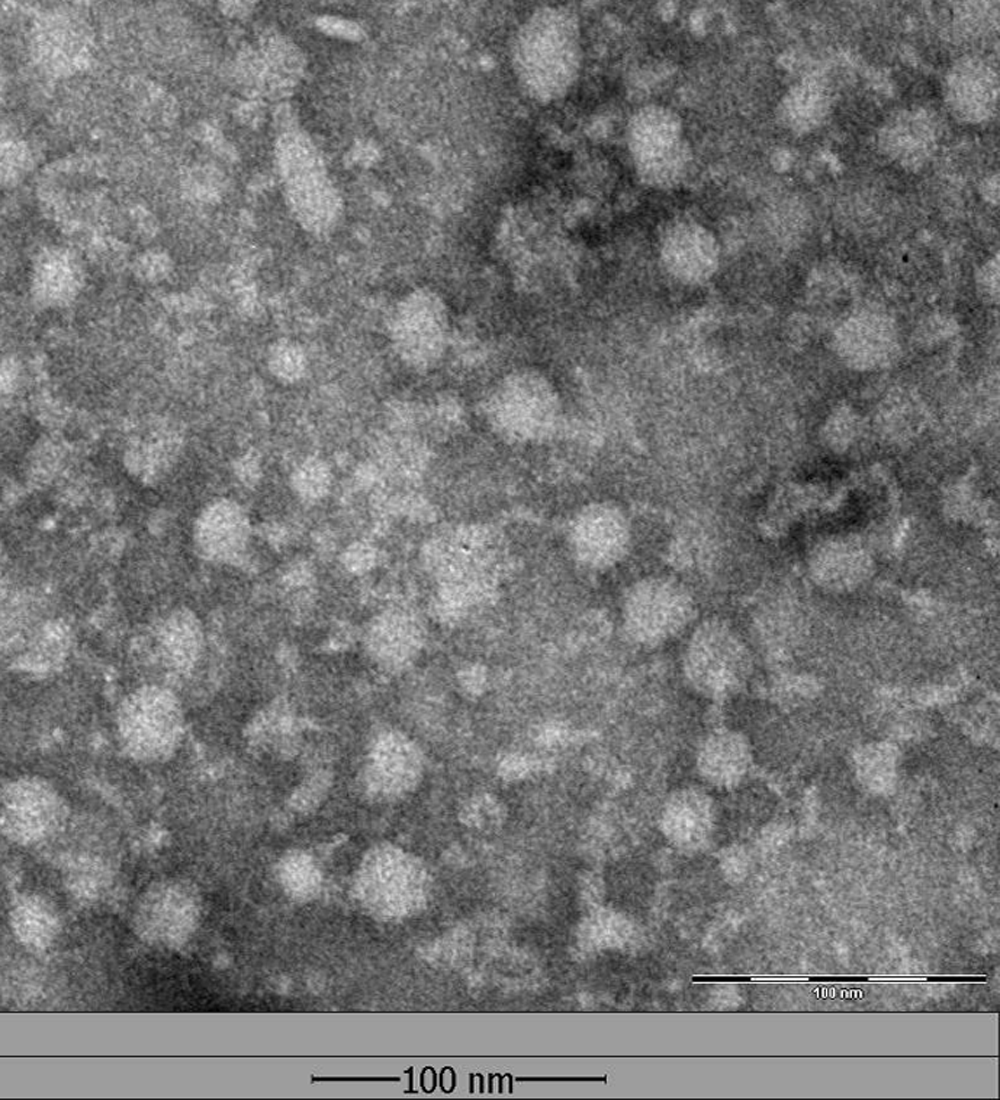

Supplement: Figure S1 — The sequence of 124-167 was inserted into the CP of MS2. But only a few chimeric proteins were obtained, which could not be assembled into nanoparticles. TEM picture as shown. [file peerj-06-4823-s005.png]
